# Efficacy and safety of IL-6 inhibitors in patients with COVID-19 pneumonia: a systematic review and meta-analysis of multicentre, randomized trials

**DOI:** 10.1186/s13613-021-00941-2

**Published:** 2021-10-26

**Authors:** Alessandro Belletti, Corrado Campochiaro, Marilena Marmiere, Valery Likhvantsev, Andrey Yavorovskiy, Lorenzo Dagna, Giovanni Landoni, Alberto Zangrillo, Ludhmila Abrahão Hajjar

**Affiliations:** 1 Department of Anesthesia and Intensive Care, IRCCS San Raffaele Scientific Institute, Milan, Italy; 2 Unit of Immunology, Rheumatology, Allergy and Rare Diseases, IRCCS San Raffaele Scientific Institute, Milan, Italy; 3 I.M. Sechenov First Moscow State Medical University (Sechenov University), Moscow, Russia; 4 Federal Research and Clinical Center of Resuscitation and Rehabilitation, Moscow, Russia; 5 School of Medicine, Vita-Salute San Raffaele University, Milan, Italy; 6 Department of Cardiopneumology, Instituto Do Coração, Universidade de São Paulo, Av. Dr. Enéas de Carvalho Aguiar 44, São Paulo, 05403-900 Brazil

**Keywords:** COVID-19, Immunomodulation, Tocilizumab, Hyperinflammation, MicroCLOTS, Acute respiratory distress syndrome

## Abstract

**Purpose:**

COVID-19 is characterized by dysregulated immune response, respiratory failure and a relevant mortality rate among hospitalized patients. Interleukin-6 (IL-6) is involved in COVID-19-associated cytokine storm, and several trials investigated whether its inhibition could improve patients’ outcome. We performed a meta-analysis of randomized trials (RCT) to test this hypothesis.

**Materials and methods:**

Two independent investigators searched PubMed, Scopus, ClnicalTrials.gov and medRxiv up to September 1st, 2021. Inclusion criteria were: administration of tocilizumab or sarilumab; COVID-19 adult patients with pneumonia; and being a RCT. Primary outcome was mortality at the longest follow-up. Secondary outcomes included intubation rate and incidence of adverse events. Two independent investigators extracted data from eligible trials.

**Results:**

Of the 763 studies assessed, 15 RCTs were included (9,320 patients), all were multicentre, and the majority open-label vs standard treatment. IL-6 inhibitors were associated with reduced all-cause mortality at the longest follow-up (1315/5,380 [24.4%] in the IL-6 inhibitors group versus 1080/3,814 [28.3%] in the control group, RR = 0.90; 95% CI 0.84 to 0.96; *p* for effect = 0.003, *I*^2^ = 0%, with 13 studies included), with reduction in 28/30-day mortality and intubation rates, and with no increase in adverse events and secondary infections.

**Conclusion:**

IL-6 inhibitors reduced longest follow-up mortality and intubation in COVID-19 patients. Findings need to be confirmed in high-quality RCTs.

**Supplementary Information:**

The online version contains supplementary material available at 10.1186/s13613-021-00941-2.

## Introduction

In December 2019 a novel coronavirus outbreak due to Severe Acute Respiratory Syndrome Coronavirus 2 (SARS-CoV-2) emerged in China [1] This virus was linked to a new emerging respiratory disease named Coronavirus disease 2019 (COVID-19) associated with significant mortality and morbidity which spread worldwide over the months and was declared a pandemic by the World Health Organization in March 2020 [2]. From the very beginning, patients affected by severe COVID-19 were found to have an uncontrolled inflammatory response resembling that observed in patients with cytokine-release syndrome [3]. Among several cytokines, serum levels of interleukin-6 (IL-6) were associated with severity of the clinical manifestations and poor outcome of COVID-19 patients [4, 5]. Consequently, given the absence of SARS-CoV-2-specific therapies, the monoclonal antibody tocilizumab, which specifically targets the IL-6 receptor and is currently approved for the treatment of rheumatoid arthritis and giant cell arteritis [6, 7], and the monoclonal antibody sarilumab, which also targets the IL-6 receptor and is approved for the treatment of rheumatoid arthritis [8], were evaluated for the moderate–severe group of patients [9]. As a consequence, several retrospective studies evaluated the role of tocilizumab in severe COVID-19 patients over the last months, with contradictory results [10–12]. Thanks to an unpreceded effort, randomized-controlled trials (RCTs) were rapidly set up and carried out in several countries to properly investigate the role of tocilizumab in COVID-19.

Given the urgent need for effective treatments for COVID-19 and the social and economic costs associated with the pandemic, we perform a systemic review of all published RCTs to answer to the unsolved question: should we use IL-6 inhibitors to treat our COVID-19 patients?

## Materials and methods

The present systematic review and meta-analysis of RCTs was performed in accordance with Preferred Reporting Items for Systematic Reviews and Meta-Analyses (PRISMA) guidelines and following Cochrane Collaboration recommendations [13–17]. The review was registered on International Prospective Register of Systematic Reviews (PROSPERO) under registration no. CRD42021230944 on January 18th 2021.

### Search strategy and study selection

Randomized controlled trials comparing tocilizumab or sarilumab versus any comparator (placebo, standard treatment, or active comparator) were included in this study. The following PICOS criteria were followed: population—patients with COVID-19 pneumonia; interventions—tocilizumab, sarilumab; comparison intervention—placebo; standard treatment; any active comparator; outcome—longest follow-up mortality, secondary outcomes as described below; study design—randomized-controlled trials. No exclusion by publication date or language was enforced.

In detail, we included studies with all the following criteria: administration of tocilizumab or sarilumab; in adult patients with COVID-19 pneumonia; and randomized-controlled trials. Exclusion criteria were (at least one of the following): overlapping population; non-randomized trials; setting other than COVID-19 pneumonia; paediatric studies; non-human studies; lack of data for outcomes of interest; studies directly comparing tocilizumab against steroids [18]; and studies published as abstract only.

We searched PubMed, Scopus, ClinicalTrials.gov and medRxiv databases. Search was performed independently by two trained investigators for suitable articles, and last updated on September 1^st^, 2021. We applied backward snowballing to retrieve additional manuscripts. Eligibility assessment was first performed by two independent investigators at title/abstract level. Subsequently, the final selection of included articles was based on the complete manuscripts and performed by two independent investigators, with disagreements solved by consensus. The search strategies are available in the Additional file 1.

### Data extraction

Details on baseline characteristics (setting, methodological details, number of patients in ICU, number of patients on invasive mechanical ventilation, number of patients receiving steroids), procedural (study drug dose, timing and mode of administration, blinding) and outcome data were independently collected by two investigators. Divergences were resolved by consensus. Data were extracted following the intention-to-treat principle whenever possible.

Corresponding authors of individual studies were contacted by email to obtain further data.

### Outcomes

The primary outcome was mortality at the longest follow-up available. Secondary outcomes were: 28/30-day mortality, need for intubation, clinical worsening, number of patients with at least one serious adverse event, number of patients with at least one secondary infection. Outcomes were defined according to individual studies author’s definition, and definitions are provided in the Additional file 1.

### Statistical analysis

For dichotomous outcomes, we calculated individual and pooled risk ratio (RR) with 95% confidence intervals (CI). For continuous variables, mean difference (MD) or standardized mean difference (SMD) with corresponding 95% CI were calculated. If necessary, continues variables were converted into mean and standard deviation following the methodology described by Wan et al. [19].

Heterogeneity analysis was performed with Cochran Q statistic and quantified with *I*^2^. Heterogeneity with an *I*^2^ > 25% was considered significant. We employed the fixed effect model or the random-effects model in case of low or high statistical heterogeneity, respectively.

Publication bias for primary endpoint was assessed by visual inspection of funnel plot if the number of retrieved studies was greater than 10 [20].

Risk of bias was assessed according to a modified version of the Risk-of-Bias 2 tool of the Cochrane Collaboration [17, 21]. Two trained investigators evaluated each item and provided an overall judgement of low risk, high risk, some concerns or unclear risk of bias. Trials with published results that did not underwent peer-review [22], single-centre trials [23, 24], and open-label trials [25], were considered to have at least “some concerns” of bias.

We planned the following pre-specified subgroup analyses: patients in ICU, patients on mechanical ventilation, studies with a high prevalence (> 50% of patients) of concomitant steroid therapy. However, we found only one study enrolling patients in the ICU, and no studies enrolling only patients on invasive mechanical ventilation. Therefore, the subgroup analysis on studies enrolling ICU patients was not performed. For the invasive mechanical ventilation subgroup analysis, we analysed studies including also patients on mechanical ventilation at baseline. In addition, we performed an unplanned subgroup analysis of patients who received versus those who did not receive steroids in addition to IL-6 inhibitors.

The following sensitivity analyses were performed: low risk of bias trials only; sequential removal of each individual trial and re-analysis of the remaining dataset; change of analysis methods; change of summary statistics, and analysis excluding patients receiving sarilumab.

For pooled outcome analyses, a p-value less than 0.05 was considered significant. Analysis was performed using RevMan 5.4. software (Review Manager, The Nordic Cochrane Centre, The Cochrane Collaboration, Copenhagen, Denmark).

Trial sequential analysis (TSA) was performed [26, 27]. We performed a fixed-effects TSA with the intent of maintaining an overall 5% risk of type I error and a 20% risk of type II error, at a power of 80%. We assumed a relative risk reduction of 15% and derived the control event proportion from the dataset. The resulting required information size was further diversity (*D*2)-adjusted. In the case of *D*2 = 0, we performed a sensitivity analysis assuming a *D*2 = 25%. The TSA Viewer software was used to perform TSA (TSA Viewer [Computer program], version 0.9.5.5 Beta, Copenhagen Trial Unit, Centre for Clinical Intervention Research, Rigshospitalet, 2016).

## Results

**Fig. 1:**
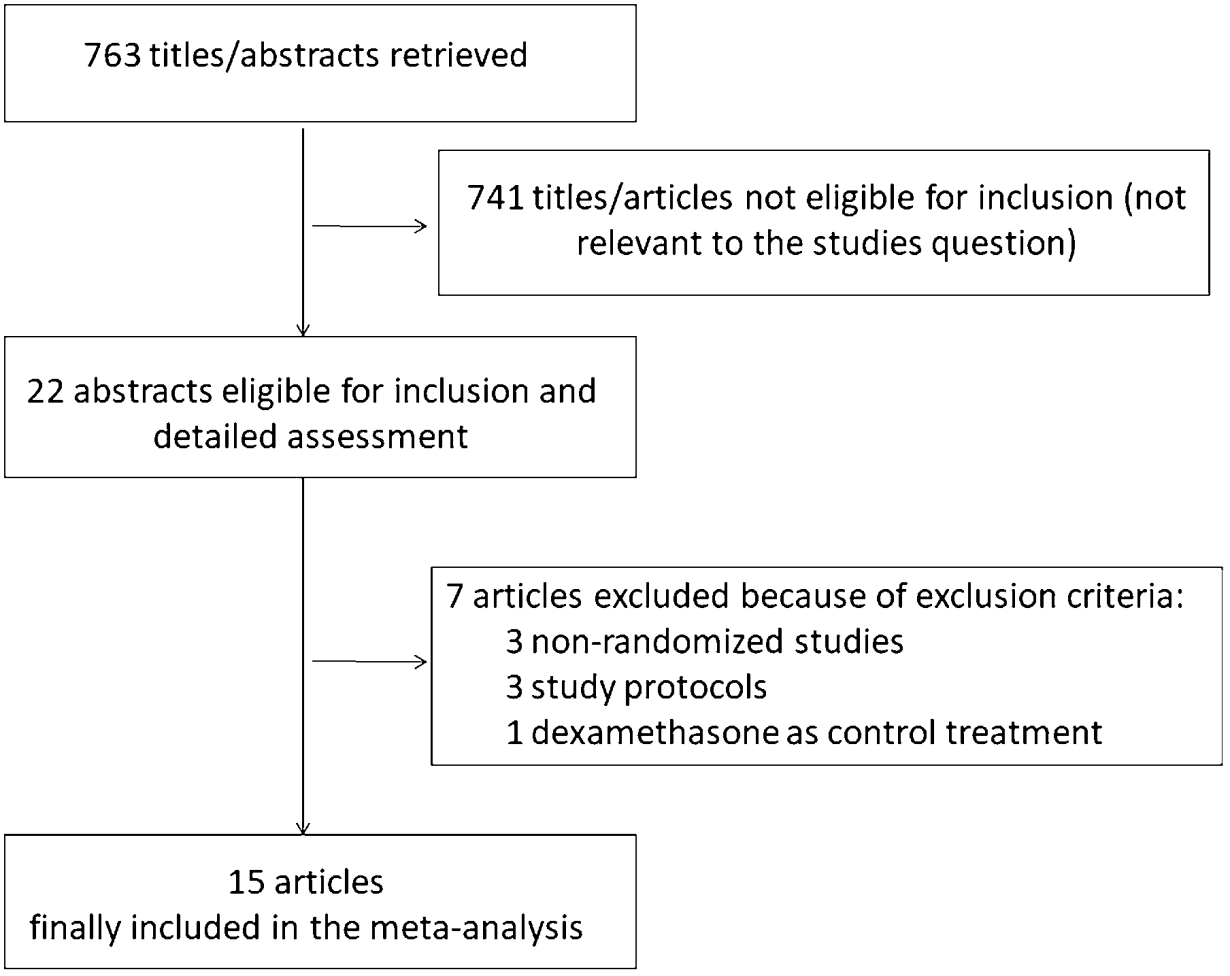
Flow diagram for study inclusions

A total of 763 references were examined at a title/abstract level. After initial screening, a total of 22 studies were retrieved as complete articles. After exclusion of three non-randomized trials [28–30], three study protocols [31–33], and one study comparing tocilizumab against dexamethasone [34], a total of 15 studies randomizing 9320 patients were included in the analysis (Fig. 1) [35–49].

### Characteristics of included trials

Characteristics of included trials are reported in Table 1 and Additional file 1: Table S2. All studies were multicentre. Three studies have not yet undergone peer-review as of September 15th 2021 and are available as pre-print only [42, 44, 47]. One study was performed on ICU patients alone [48], and eight studies also included patients under invasive mechanical ventilation at baseline [34, 40, 41, 43, 45–48] (Table 2). Concomitant use of steroids was highly variable across studies, ranging from 10.6 to 91.1% of enrolled patients (Table 2). Seven studies specifically enrolled patients with documented hyperinflammation [35, 36, 39, 42–44, 49], including two studies enrolling patients with documented elevated IL-6 [39, 42], and one study enrolling patients with elevated C-reactive protein (CRP) [43] (Table 1).

**Table 1:** Characteristics of included studies

First author	Acronym	Journal	Setting	Trial registration number	Blinding	Treatment	Control
Gordon AC	REMAP-CAP	N Engl J Med	COVID-19 critically ill patients	ClinicalTrials.gov NCT02735707	Open-label	Tocilizumab, sarilumab	Standard care
Hermine O	CORIMUNO-TOCI 1	JAMA Intern Med	COVID-19 moderate/severe pneumonia	ClinicalTrials.gov NCT04331808	Open-label	Tocilizumab	Standard care
Lescure FX	N/A	Lancet Respir Med	COVID-19 severe pneumonia	ClinicalTrials.gov NCT04327388	Double-blind	Sarilumab	Placebo
RECOVERY Collaborative Group	RECOVERY	Lancet	COVID-19 pneumonia (SpO_2_ < 92%), elevated CRP	ClinicalTrials.gov NCT04381936	Open-label	Tocilizumab	Standard care
Rosas IO	COVACTA	N Engl J Med	COVID-19 severe pneumonia	ClinicalTrials.gov NCT04320615	Double-blind	Tocilizumab	Placebo
Rutgers A	PreToVid	SSRN Electron J	COVID-19 pneumonia with hyperinflammation	Netherlands Trial Register NL8504	Open-label	Tocilizumab	Standard care
Salama C	EMPACTA	N Engl J Med	COVID-19 pneumonia, not on NIMV/IMV	ClinicalTrials.gov NCT04372186	Double-blind	Tocilizumab	Placebo
Salvarani C	RCT-TCZ-COVID-19	JAMA Intern Med	COVID-19 pneumonia, hyperinflammatory state, not on MV, not in ICU	ClinicalTrials.gov NCT04346355	Open-label	Tocilizumab	Standard care
Sivapalasingam S	N/A	medXriv	COVID-19 pneumonia requiring supplemental oxygen	ClinicalTrials.gov NCT04315298	Double-blind	Sarilumab	Placebo
Soin AS	COVINTOC	Lancet Respir Med	COVID-19 moderate-to-severe pneumonia	Clinical Trials Registry India CTRI/2020/05/025369	Open-label	Tocilizumab	Standard care
Stone JH	BACC Bay Tocilizumab Trial	N Engl J Med	COVID-19 pneumonia, hyperinflammatory state, not on MV	ClinicalTrials.gov NCT04356937	Double-blind	Tocilizumab	Placebo
Talaschian M	N/A	Research Square	COVID-19 pneumonia with elevated CRP/IL-6, not on IMV	Iranian Registry of Clinical Trials IRCT20081027001411N4	Open-label	Tocilizumab	Standard care
Veiga VC	TOCIBRAS	BMJ	COVID-19 severe/critical pneumonia	ClinicalTrials.gov NCT04403685	Open-label	Tocilizumab	Standard care
Wang D	N/A	SSRN Electron J	COVID-19 moderate/severe pneumonia, elevated IL-6	Chinese Clinical Trial Registry ChiCTR2000029765	Open-label	Tocilizumab	Standard care
Zhao H	N/A	Biomed Pharmacother	COVID-19 pneumonia, elevated IL-6	ClinicalTrials.gov NCT04310228	Open-label	Tocilizumab	Favipiravir

COVID-19: coronavirus disease 2019; CRP: C-reactive protein; IL-6: interleukin-6; IMV: invasive mechanical ventilation; MV: mechanical ventilation; N/A: not available; NIMV: non-invasive mechanical ventilation; SpO_2_: peripheral oxygen saturation

**Table 2:** Treatment characteristics of included trials

First author	Acronym	Treatment dose	Control dose	Time from symptoms onset to first dose, days	Patients in ICU at baseline, %	Patients on IMV at baseline, %	Patients who received steroids, %	Primary outcome	Longest follow-up
Gordon AC	REMAP-CAP	Tocilizumab: 8 mg/kg IV (max 800 mg); second dose 12–24 h at physician discretion; Sarilumab: 400 mg IV, one dose	N/A	N/A	100.0	29.0	90.7	Respiratory and cardiovascular organ support-free days up to day 21	Hospital stay
Hermine O	CORIMUNO-TOCI 1	8 mg/kg IV (max 800 mg); second dose 400 mg on day 3 at discretion of attending physician	N/A	10	0.0	0.0	47.7	Death or NIMV/IMV day 4 (WHO-CPS > 5); survival without MV on day 14	Hospital stay
Lescure FX	N/A	400 mg IV or 200 mg IV. Second dose based on clinician’s discretion within 24–48 h	N/A	5	35.6	11.6	42.1	Time to clinical improvement	60 days
RECOVERY Collaborative Group	RECOVERY	800 mg if weight > 90 kg; 600 mg if weight > 65 and ≤ 90 kg; 400 mg if weight > 40 and ≤ 65 kg; and 8 mg/kg if weight ≤ 40 kg. A second dose could be given, according to the clinician opinion, if patient’s condition did not improve	N/A	9 (tocilizumab) vs 10 (standard care)	N/A	13.7	82.2	28-day mortality	28 days
Rosas IO	COVACTA	8 mg/kg IV (max 800 mg); second dose 8–24 h after if no improvement/worsening	N/A	11 (tocilizumab) vs 10 (placebo)	56.4	37.7	42.2	Clinical status on 7-category ordinal scale at day 28	28 days
Rutgers A	PreToVid	8 mg/kg IV (max 800 mg); second dose 8 h after if hypoxia not resolved	N/A	9 (tocilizumab) vs 9 (standard care)	N/A	N/A	88.4	30-day mortality	30 days
Salama C	EMPACTA	8 mg/kg IV (max 800 mg); second dose 8–24 h after if no improvement/worsening	N/A	8	15.4	0.0	82.8	Death or IMV/ECMO by day 28	60 days
Salvarani C	RCT-TCZ-COVID-19	8 mg/kg IV (max 800 mg), 2 doses 12 h apart	N/A	8	0.0	0.0	10.6	Clinical worsening within 14 days	Hospital stay
Sivapalasingam S	N/A	200 mg IV, 400 mg IV, or 800 mg IV	N/A	N/A	N/A	N/A	29.0	CRP levels (Phase 2); Clinical improvement in patients receiving MV at baseline (Phase 3)	29 days
Soin AS	COVINTOC	6 mg/kg IV (max 480 mg); second dose 12 h–7d after if no improvement/worsening	N/A	N/A	65.9	5.0	91.1	Disease progression (from moderate to severe or from severe to death) within 14 days	Hospital stay
Stone JH	BACC Bay Tocilizumab Trial	8 mg/kg IV (max 800 mg)	N/A	9	4.1	0.0	11.6	Death or IMV by day 28	28 days
Talaschian M	N/A	8 mg/kg IV (max 800 mg)	N/A	N/A	0.0	0.0	33.3	28-day mortality	28 days
Veiga VC	TOCIBRAS	8 mg/kg IV (max 800 mg)	N/A	10	N/A	16.3	86.0	Clinical status at day 15 (seven-level ordinal scale)	29 days
Wang D	N/A	400 mg IV; second dose 24 h if still fever	N/A	23	N/A	0.0	N/A	Cure rate of enrolled patients	Hospital stay
Zhao H	N/A	4–8 mg/kg IV; second dose 24 h if still fever	1600 mg × 2 p.p. day 1; 600 mg × 2 p.o. day 2–7	N/A	N/A	0.0	N/A	Cumulative lung lesion remission rate (lung CT examination indicated absorption of lung inflammation)	Hospital stay

CT: computed tomography; ECMO: extracorporeal membrane oxygenation; ICU: intensive care unit; IMV: invasive mechanical ventilation; IV: intravenous; N/A: not available; NIMV: non-invasive mechanical ventilation; WHO-CPS: World Health Organization Clinical Performance Scale

Five studies compared IL-6 inhibitors with placebo [36, 38, 40, 45, 47], nine studies with standard treatment, [35, 37, 41–44, 46, 48, 49] and one study against favipiravir [42].

Overall, risk of bias analysis showed that three of included trials were at low risk of bias [36, 38, 40], while the remaining had at least some concerns of bias, mainly due to lack of blinding or lack of peer-review (Additional file 1).

### All-cause mortality

**Fig. 2:**
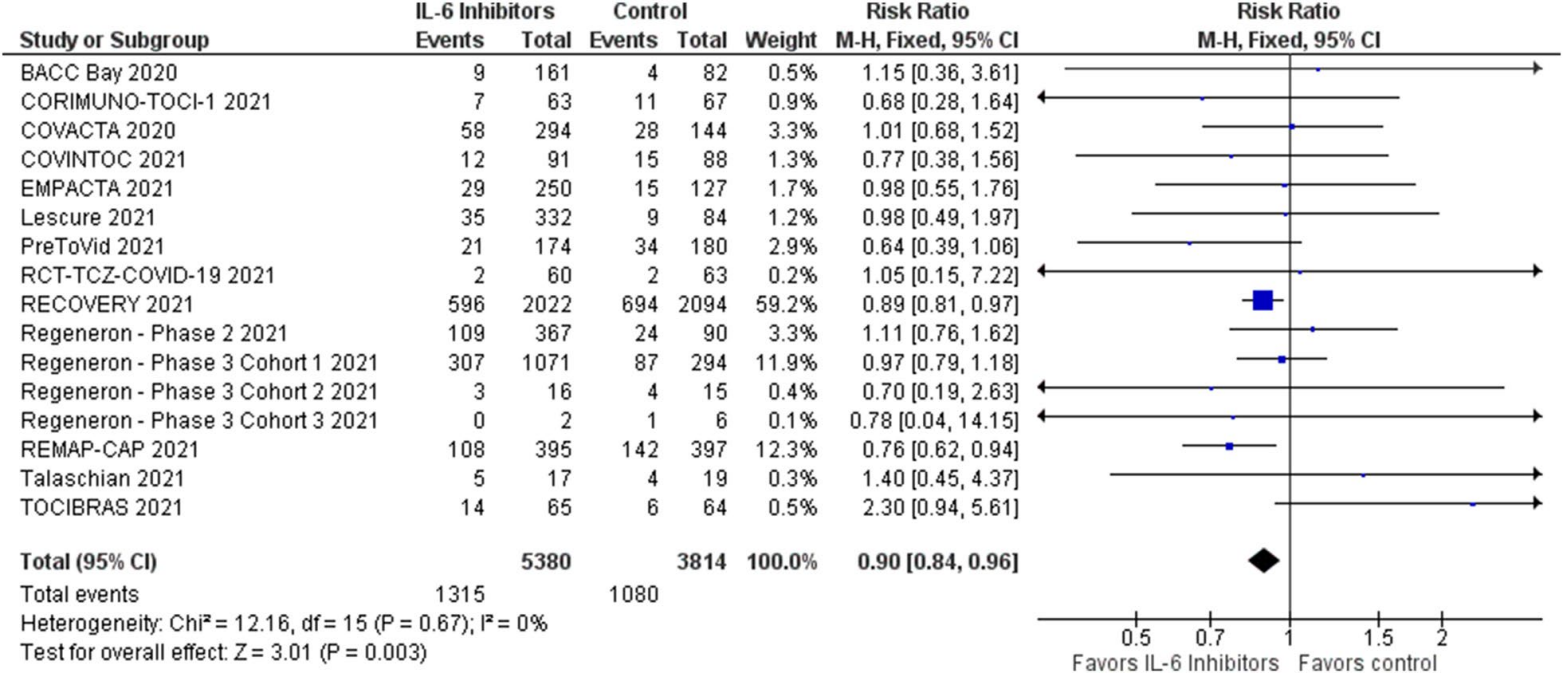
Forest plot for longest follow-up mortality

Overall, we found that IL-6 inhibitors administration was associated with a significant reduction in all-cause longest follow-up mortality (1315/5380 [24.4%] in the IL-6 inhibitors group versus 1080/3814 [28.3%] in the control group, RR = 0.90; 95% CI 0.84 to 0.96; *p* for effect = 0.003, *I*^2^ = 0%, with 13 studies included; NNT = 26) (Fig. 2).

Changes of the summary statistics from RR to odds ratio or risk difference did not result in a change in significance of study findings. Similarly, changing from fixed- to random-effects model did not alter significance of the results (RR for random-effects model = 0.89; 95% CI = 0.83 to 0.96; *p*-value = 0.002).

Sequential removal of each trial showed that statistical significance is lost when removing the RECOVERY study (RR without RECOVERY = 0.91; 95% CI 0.81 to 1.02; *p*-value = 0.09; *I*^2^ = 0%) [43].

Analyses including the three low risk of bias studies only, suggested the lack of benefit of IL-6 inhibitors (longest follow-up mortality was 96/705 [13.6%] in the IL-6 inhibitors group versus 47/453 [10.3%] in the control group, RR = 1.01; 95% CI 0.74 to 1.40; *p*-value = 0.93; *I*^2^ = 0%, with three trials included).

Analysis excluding patients receiving sarilumab confirmed magnitude and direction of the results (851/3,547 [24.0%] in the tocilizumab group versus 955/3325 [28.7%] in the control group, RR = 0.88; 95% CI 0.81 to 0.95; *p* for effect = 0.001, *I*^2^ = 0%, with 11 studies included) (Additional file 1).

**Fig. 3:**
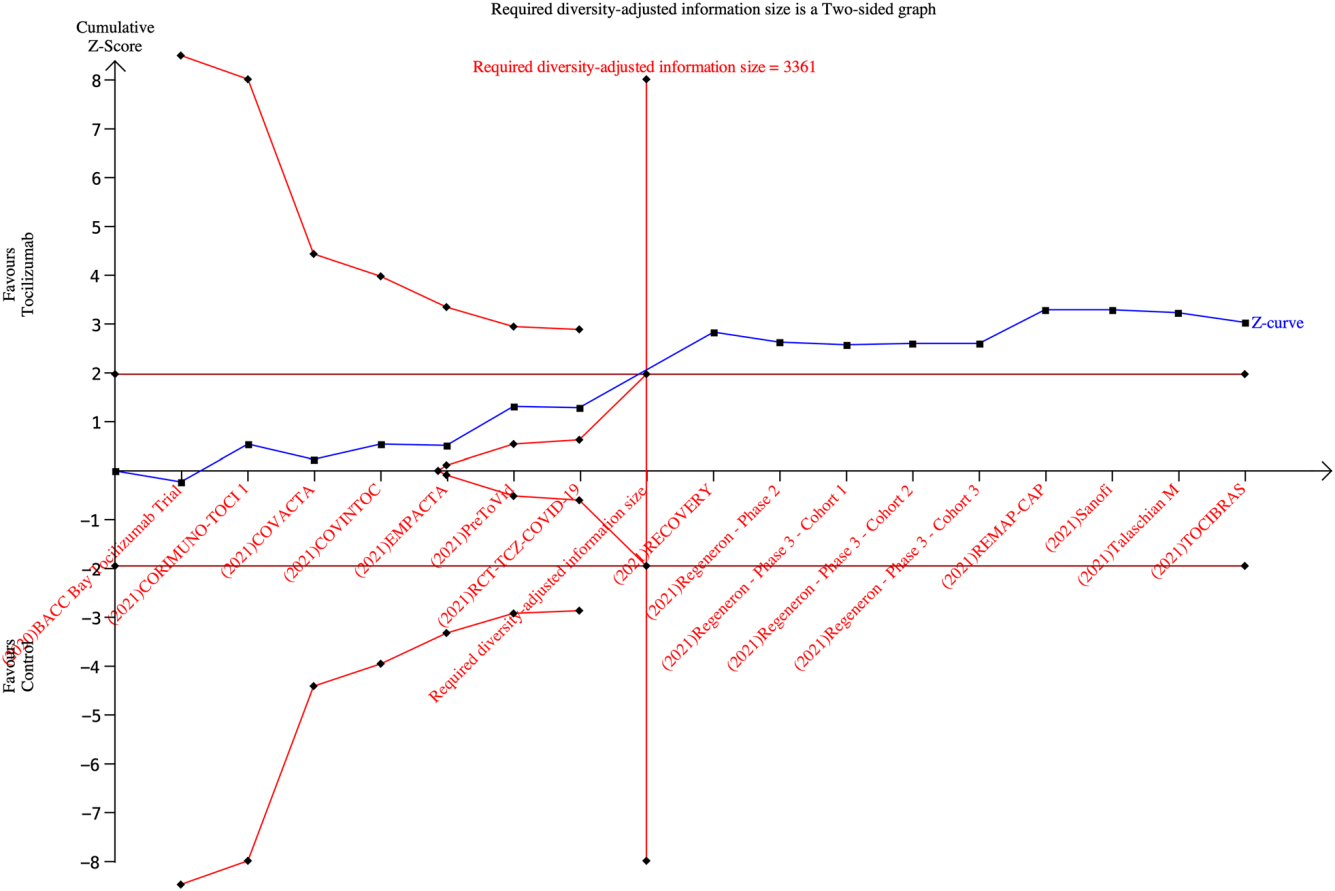
Trial sequential analysis for primary outcome

Interleukin-6 inhibitors reduced mortality significantly compared to controls according to TSA (TSA-adjusted 95% CI = 0.81 to 0.99; required information size = 3,361). In particular, TSA showed that the cumulative Z curve crossed boundaries for benefit, suggesting that information size has been reached (Fig. 3). Trial sequential analysis results were confirmed at sensitivity analysis assuming a *D*2 = 25% (Additional file 1).

Visual inspection of funnel plot did not suggest presence of publication bias (Additional file 1).

### Secondary outcomes

Results of secondary outcome analyses are presented in the Additional file 1. A total of 11 trials reported 28/30-day mortality data with a significant improvement in survival in the IL-6 inhibitors group (1193/4,967 [24%] in the IL-6 inhibitors group versus 924/3399 [27.1%] in the control group, RR = 0.92; 95% CI 0.85 to 0.99; *p* = 0.03, *I*^2^ = 0%).

**Fig. 4:**
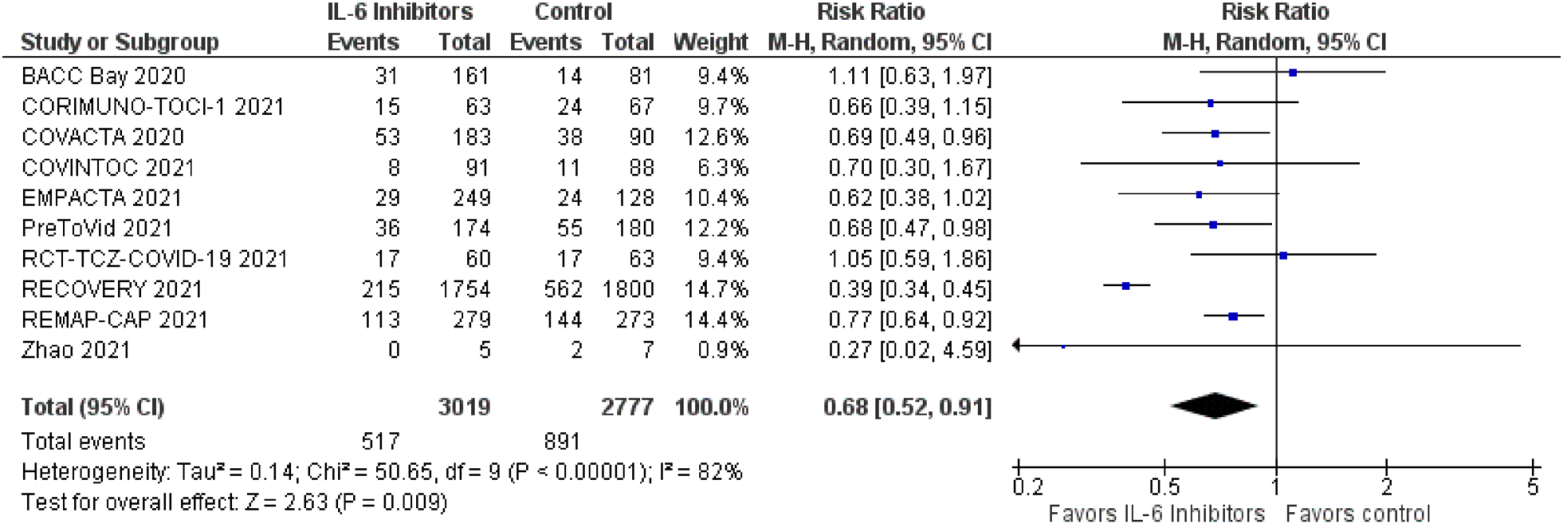
Forest plot for clinical worsening

In addition, we found that use of IL-6 inhibitors was associated with a significant reduction in need for intubation (171/1933 [8.8%] versus 180/1649 [10.9%]; RR = 0.73; 95% CI = 0.60 to 0.88; *p* = 0.001; *I*^2^ = 0%; 9 trials included) and clinical worsening (517/3,019 [17.1%] versus 891/2777 [32.0%]; RR = 0.68; 95% CI = 0.52 to 0.91; *p* = 0.009; *I*^2^ = 82%; 10 trials included) (Fig. 4).

We observed no significant difference in occurrence rate of serious adverse events (983/5166 [19%] versus 354/3604 [9.8%]; RR = 1.00; 95% CI = 0.90 to 1.10; *p* = 0.99; *I*^2^ = 1%; 13 trials included), and occurrence rate of secondary infections (177/1593 [11.1%] versus 130/1259 [10.3%]; RR = 0.78; 95% CI = 0.60 to 1.01; *p* = 0.06; *I*^2^ = 11%; 11 trials included).

When analysing low risk of bias studies only, the need for intubation was reduced in the IL-6 inhibitors group (82/593 [13.8%] versus 57/299 [19.1%]; RR = 0.72; 95% CI = 0.53 to 0.96; *p* = 0.03; *I*^2^ = 0%; 3 trials included), as well as clinical worsening (113/593 [19.1%] versus 76/299 [25.4%]; RR = 0.74; 95% CI = 0.55 to 1.00; *p* = 0.05; *I*^2^ = 25%, 3 trials included) (Additional file 1). For all other outcomes, we observed no significant differences.

### Subgroup analyses

Results of subgroup analyses of studies enrolling vs not enrolling patients on invasive mechanical ventilation at baseline are presented in the Additional file 1. We found a significant subgroup interaction when analysing the effect of IL-6 inhibitors on the rate of secondary infections, with studies not enrolling patients on invasive mechanical ventilation showing benefit as compared with studies also enrolling patients on invasive mechanical ventilation (*p* for interaction = 0.04) (Additional file 1).

Three trials reported longest follow-up and 28/30-day mortality data stratified by steroids use [40, 43, 47]. In this analysis, we found a significant subgroup interaction, with improved survival in patients receiving steroids together with IL-6 inhibitors (*p* for interaction = 0.004) (Additional file 1). In the other subgroup analyses by concomitant use of steroids, we found no significant subgroup interactions (Additional file 1).

## Discussion

### Key findings

In this meta-analysis of multicentre RCTs, we found that IL-6 inhibitors administration in patients with COVID-19 pneumonia is associated with a significant reduction in longest follow-up mortality. Furthermore, it is associated with reduction in 28/30-day mortality, need for intubation, and clinical worsening. The beneficial effect on the need for intubation and clinical worsening was confirmed also when analysing studies with a low risk of bias. Results on mortality are largely driven by the RECOVERY trial. [43].

### Relationship with previous studies

Several meta-analyses and systematic review on this topic have been recently published [18, 50–60]. However, most of these meta-analyses did not include the most recently published trials such as the RECOVERY trial [43].

The largest meta-analysis published so far by the World Health Organization (WHO) Rapid Evidence Appraisal for COVID-19 Therapies (REACT) Working Group included a total 27 randomized studies, 18 of which were not published at time of the meta-analysis publication. They found that IL-6 inhibition was associated with improved 28-day survival and reduction in the composite endpoint of need for invasive mechanical ventilation, extracorporeal membrane oxygenation, or death. Our study also found a reduction in 28/30-day mortality, together with a reduction in need for invasive mechanical ventilation and in a composite endpoint of clinical worsening. Compared with the WHO REACT meta-analysis, our study focused on a different primary endpoint (longest follow-up mortality) and on studies with published results (even if not peer-reviewed). Furthermore, due to different search strategy, inclusion criteria and last update, we included four trials not included in the WHO REACT meta-analysis [35, 39, 42, 44]. There are also some methodological difference in the statistical analysis. For example, we used a Mantel–Haenszel weighted risk ratio versus an inverse variance-weighted odds ratio. However, analysis of our data using a similar approach did not lead to change in magnitude and direction of results. We also analysed risk of bias using somewhat more restrictive criteria for unpublished, open-label and single-centre trials, and therefore our risk of bias analysis results in a lower proportion of low risk of bias studies [22–25]. Finally, we also performed a TSA. Overall, despite these methodological differences, we believe that our work is complementary to the WHO REACT study and that it confirms the potential beneficial effect of IL-6 inhibitors in patients with COVID-19 also found by the WHO REACT colleagues. In particular, our study suggest that IL-6 inhibitors benefit may extend beyond 28/30 days, and our TSA showed that the required information size was reached, suggesting firm evidence on beneficial effect of IL-6 inhibitors.

### Significance of study findings and what this study adds to our knowledge

Our meta-analysis provides some evidence that tocilizumab administration may be beneficial in patients with COVID-19 pneumonia, by reducing the risk of death and the risk of intubation without increasing risk of secondary infections and adverse events.

Most of the studies enrolled patients about 10 days after symptoms onset, with a moderate–severe disease. Only one RCT was specifically performed in ICU patients [48], and several studies excluded patients requiring mechanical ventilation at baseline. Therefore, we are unable to comment on efficacy and safety of IL-6 inhibitors in critically ill patients and particularly on those receiving mechanical ventilation, which may have the highest risk of secondary infections [61–63].

Nevertheless, the REMAP-CAP study (the only study entirely performed in an ICU setting) is one of the two studies showing a significant improvement in patients survival without higher rate of severe infections [48]. However, less than 30% of patients enrolled in the REMAP-CAP were receiving invasive mechanical ventilation at baseline.

Another interesting finding of our systematic review is that approximately half of included studies did not screen patients on the basis of baseline elevated inflammatory markers [37, 38, 40, 41, 48], including the REMAP-CAP trial [48]. However, the RECOVERY trial enrolled patients with elevated baseline C-reactive protein, somehow supporting the rationale of administering tocilizumab to patients with documented inflammation [43]. Moreover, in accordance with the results of the RECOVERY trial and WHO REACT meta-analysis [18], we also observed that the concomitant use of steroids and IL-6 inhibitors was associated with a significant reduction in clinical worsening.

In addition, we also explored the risk of development of secondary infections as well as serious adverse events and found no differences between patients receiving IL-6 inhibitors and controls, suggesting safety of this therapy.

Notably, only five trials were double-blinded [36, 38, 40, 45, 47] and only three were judged to carry a low risk of bias [36, 38, 40]. All others RCTs were open-label. Therefore, we believe that the overall treatment effect of IL-6 inhibitors may be overestimated, as it has been showed that blinded trials generally have a 40% higher NNT when compared to unblinded trials [25]. Nevertheless, we do acknowledge that the ongoing pandemic and the pressure on most healthcare systems prompted the need to test therapies in a short timeframe, thereby not allowing in most cases to organize double-blind placebo-controlled trials.

Notably, the reduction in need for invasive mechanical ventilation as well as in clinical worsening were confirmed also when analysing double-blind, low risk of bias studies.

Collectively, results of our meta-analysis suggest that administration of IL-6 inhibitors may be beneficial in patients in a relatively early stage of the disease not undergoing invasive mechanical ventilation and treated together with systemic steroids. Although further high-quality evidence is required, use of IL-6 inhibitors may be justified in a context of pandemic and high pressure on healthcare systems and intensive care units [64, 65].

### Strengths and limitations of the study

Our meta-analysis includes only multicentre RCTs, thereby with highest internal and external validity, and therefore carry the highest level of evidence [23, 24, 66, 67]. Furthermore, we explored both safety and efficacy outcomes, and investigated possible subgroup interactions including concomitant use of steroids.

However, our results are limited by the overall high risk of bias of available studies and heterogeneity among inclusion criteria and concomitant treatments. We also included trials that have not yet undergone peer-review. Nevertheless, this has been a common practice in the ongoing COVID-19 pandemic [18].

We were unable to obtain additional data from investigators, and therefore we could not perform detailed analysis on interaction between corticosteroids and IL-6 inhibitors.

Statistical significance is lost when removing the RECOVERY trial [43] from analysis. However, beneficial effect of IL-6 inhibitors on clinically relevant secondary outcomes is confirmed in several sensitivity analyses, including low risk of bias trials.

The COVID-19 pandemic is still ongoing and several treatments are under investigation. We cannot comment on possible interaction between other promising treatment strategies such as anti-virals or monoclonal antibodies and IL-6 inhibitors.

### Future studies and prospects

Our study highlighted current gaps of knowledge in IL-6 inhibitors use among patients with COVID-19. In particular, there is still insufficient evidence on efficacy and safety of IL-6 inhibitors among patients with critical disease requiring invasive mechanical ventilation. Furthermore, despite several mRCTs have been published, the quality of evidence remains low since several published trials lacked blinding [25].

Future studies should be designed as double-blinded and placebo-controlled in order to provide the greatest level of scientific rigour. Our study can provide baseline information to calculate sample size for such a future trial.

Furthermore, future studies should assess the possible interactions between available treatments for COVID-19, especially the combination of both systemic steroids and tocilizumab in specific subgroup of patients with a more pronounced inflammatory phenotype.

## Conclusions

Interleukin-6 inhibitors may reduce mortality and need for intubation in patients with COVID-19 pneumonia, when administered within 10 days since symptoms onset, especially if used concomitantly with steroids. In particular, the reduction in need for intubation and in a composite endpoint of clinical worsening was confirmed also in low risk of bias studies. However, most of published RCTs lacked blinding and significance of mortality results is lost when only low-risk of bias studies are analysed.

## Supplementary Information

**Additional file 1.** Supplementary Appendix including details on search strategies, supplementary tables S1–S2, and supplementary figures S1–S25.

## Data Availability

All data generated or analysed during this study are included in this published article and its supplementary information files.
