# Supplementary material for: Efficacy and safety of IL-6 inhibitors in patients with COVID-19 pneumonia: a systematic review and meta-analysis of multicentre, randomized trials
Source: Ann Intensive Care. 2021 Oct 26;11:152. doi: 10.1186/s13613-021-00941-2 (PMC8547720; doi:10.1186/s13613-021-00941-2)

**AdditionAL file**

**Efficacy and safety of IL-6 inhibitors in patients with COVID-19 pneumonia: a systematic review and meta-analysis of multicentre randomized trials**

A. Belletti, C. Campochiaro, M. Marmiere, V. Likhvantsev,

A. Yavorovskiy, L. Dagna, G. Landoni, A. Zangrillo, L.A. Hajjar

| **Search strategies** | page 3 |
| --- | --- |
| **Supplementary Table 1** | page 4 |
| **Supplementary Table 2** | page 5 |
| **Supplementary Figure 1** | page 6 |
| **Supplementary Figure 2** | page 7 |
| **Supplementary Figure 3** | page 8 |
| **Supplementary Figure 4** | page 9 |
| **Supplementary Figure 5** | page 10 |
| **Supplementary Figure 6** | page 11 |
| **Supplementary Figure 7** | page 12 |
| **Supplementary Figure 8** | page 13 |
| **Supplementary Figure 9** | page 14 |
| **Supplementary Figure 10** | page 15 |
| **Supplementary Figure 11** | page 16 |
| **Supplementary Figure 12** | page 17 |
| **Supplementary Figure 13** | page 18 |
| **Supplementary Figure 14** | page 19 |
| **Supplementary Figure 15** | page 20 |
| **Supplementary Figure 16** | page 21 |
| **Supplementary Figure 17** | page 22 |
| **Supplementary Figure 18** | page 23 |
| **Supplementary Figure 19** | page 24 |
| **Supplementary Figure 20** | page 25 |
| **Supplementary Figure 21** | page 26 |
| **Supplementary Figure 22** | page 27 |
| **Supplementary Figure 23** | page 28 |
| **Supplementary Figure 24** | page 29 |
| **Supplementary Figure 25** | page 30 |

**SEARCH STRATEGIES**

**PubMed search strategy**

(tocilizumab[tiab] or sarilumab[tiab] or IL-6 inhibitor*[tiab] or interleukin-6 inhibitor*[tiab]) and (randomized [ti] or randomized controlled trial [pt] or randomization [TIAB] or randomly [TIAB] or randomised prospective study [tiab] or post hoc analysis [tiab] or post hoc analyses [tiab] or random number table[tiab] or assigned receive [tiab] or cluster-randomized [tiab] or cluster randomized [tiab] or cluster-crossover randomized [tiab] or cluster crossover randomized [tiab] or cohort study [tiab] or cohort analysis [ti] or retrospective study [ti] or cross-sectional study [tiab] or case control [tiab]) and (covid-19 [tiab] or corona [tiab] or covid [tiab] or nCov [tiab] or sars [tiab]) not (survey [ti] or study protocol [ti] or case-control [ti] or rabbit [ti] or squirrel [ti] or pig [ti] or rat [ti] or animal* [ot] or animal* [ti] or rats[ti])

**Scopus search strategy**

( TITLE-ABS-KEY ( tocilizumab ) OR TITLE-ABS-KEY ( sarilumab ) AND TITLE-ABS-KEY ( coronavirus AND disease 2019 ) OR TITLE-ABS-KEY ( covid-19 ) OR TITLE-ABS-KEY ( sars-cov-2 ) AND TITLE-ABS-KEY ( randomized ) )

**ClinicalTrials.gov search strategy**

tocilizumab | Covid19

sarilumab | Covid19

**medRxiv search strategy**

(sarilumab OR tocilizumab) AND (covid-19 OR coronavirus disease 2019 OR sars-cov-2) AND randomized NOT meta-analysis

**SUPPLEMENTARY TABLES**

**eTable 1** – Definition of outcomes

| **First Author** | **Acronym** | **Journal** | **Definition of clinical worsening** |
| --- | --- | --- | --- |
| Gordon AC | REMAP-CAP | N Engl J Med | Death, IMV, or ECMO |
| Hermine O | CORIMUNO-TOCI 1 | JAMA Intern Med | HFNC/NIMV/IMV/Death by day 14 |
| RECOVERY Collaborative Group | RECOVERY | Lancet | Death, IMV, or ECMO |
| Lescure FX | N/A | Lancet Respir Med | N/A |
| Rosas IO | COVACTA | N Engl J Med | Death, need for MV, need for ICU, withdrawal during hospitalization within 28 days |
| Rutgers A | PreToVid | SSRN Electron J | Death or IMV |
| Salama C | EMPACTA | N Engl J Med | Death, IMV, or ECMO |
| Salvarani C | RCT-TCZ-COVID-19 | JAMA Intern Med | Admission to ICU with mechanical ventilation; Death from any cause; PaO2/FIO2 ratio less than 150mmHg in 1 of the scheduled arterial blood gas measurements or in an emergency measurement, confirmed within 4 hours by a second examination |
| Sivapalasingam S | N/A | medRxiv | N/A |
| Soin AS | COVINTOC | Lancet Respir Med | Progression from moderate-to-severe or from severe-to-death |
| Stone JH | BACC Bay Tocilizumab Trial | N Engl J Med | Change in ordinal clinical improvement scale |
| Talaschian M | N/A | Research Square | N/A |
| Veiga VC | TOCIBRAS | BMJ | N/A |
| Wang D | N/A | SSRN Electron J | N/A |
| Zhao H | N/A | Biomed Pharmacother | Death or MV |

ECMO = extracorporeal membrane oxygenation; HFNC = high-flow nasal cannula; ICU = intensive care unit; IMV = invasive mechanical ventilation; MV = mechanical ventilation; N/A = not applicable; NIMV = non-invasive mechanical ventilation

**eTable 2 –** Risk of Bias

| **Author** | **ACRONYM** | **Randomization process** | **Deviation from interventions** | **Missing data** | **Outcome assessment** | **Selection of the reported results** | **Other** | **Overall** |
| --- | --- | --- | --- | --- | --- | --- | --- | --- |
| Gordon AC | REMAP-CAP | Low | Some concerns | Low | Low | Low | Low | Some concerns |
| Hermine O | CORIMUNO-TOCI 1 | Low | Some concerns | Low | Low | Low | Low | Some concerns |
| Lescure FX | N/A | Low | Some concerns | Low | Low | Low | Some concerns | Some concerns |
| RECOVERY Collaborative Group | RECOVERY | Low | Some concerns | Low | Low | Low | Low | Some concerns |
| Rosas IO | COVACTA | Low | Low | Low | Low | Low | Low | Low |
| Rutgers A | PreToVid | Low | Some concerns | Low | Low | Low | Some concerns | Some concerns |
| Salama C | EMPACTA | Low | Low | Low | Low | Low | Low | Low |
| Salvarani C | RCT-TCZ-COVID-19 | Low | Some concerns | Low | Low | Low | Low | Some concerns |
| Sivapalasingam S | N/A | Low | Some concerns | Low | Low | Low | Some concerns | Some concerns |
| Soin AS | COVINTOC | Low | Some concerns | Low | Low | Low | Low | Some concerns |
| Stone JH | BACC Bay Tocilizumab Trial | Low | Low | Low | Low | Low | Low | Low |
| Talaschian M | N/A | Some concerns | High | Low | Low | Low | Some concerns | High |
| Veiga Vc | TOCIBRAS | Low | Some concerns | Low | Low | Low | Low | Some concerns |
| Wang D | Wang D | Low | High | High | Low | Low | Some concerns | High |
| Zhao H | Zhao H | Unclear | High | Unclear | Low | High | Low | High |

**SUPPLEMENTARY FIGURES**

**eFigure 1 –** Forest plot for longest follow-up mortality, high versus low-risk of bias


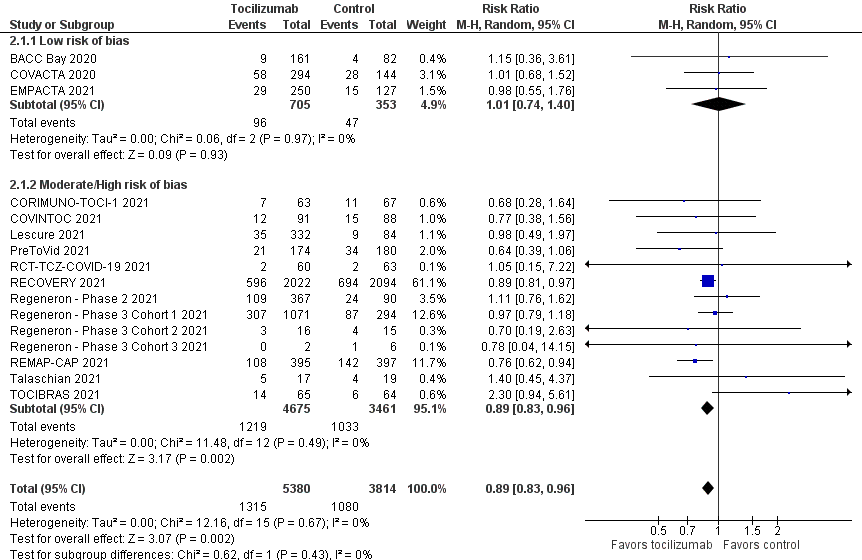


**eFigure 2 ­** Forest plot for longest follow-up mortality, excluding patients receiving sarilumab


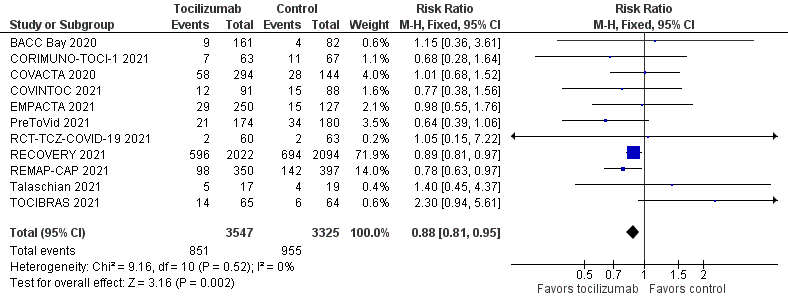


**eFigure 3 –** Trial sequential analysis for primary outcome, sensitivity analysis assuming a D2=25%


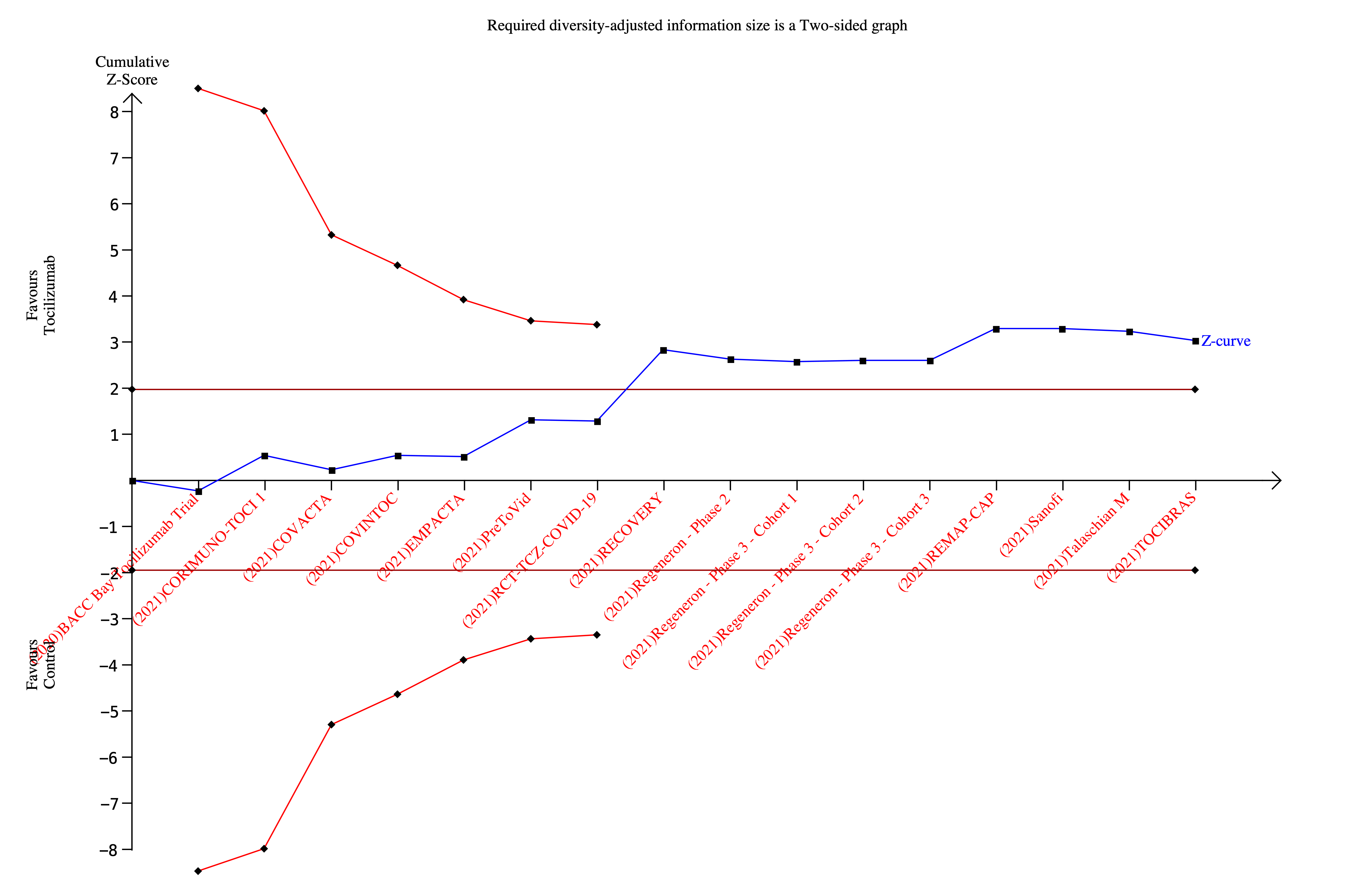


**eFigure 4 –** Funnel plot for primary outcome


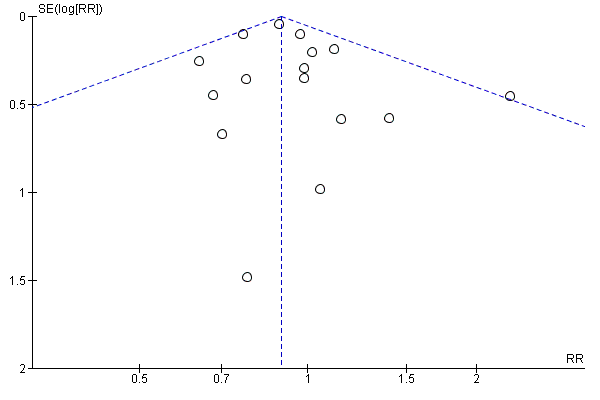


**eFigure 5 –** Forest plot for 28/30-days mortality


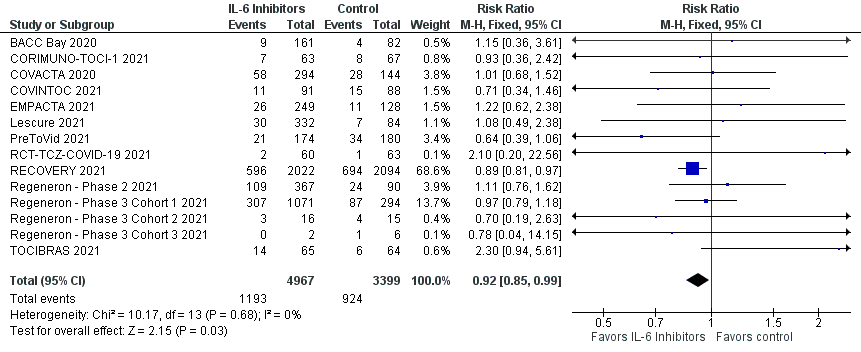


**eFigure 6 –** Forest plot for need for intubation


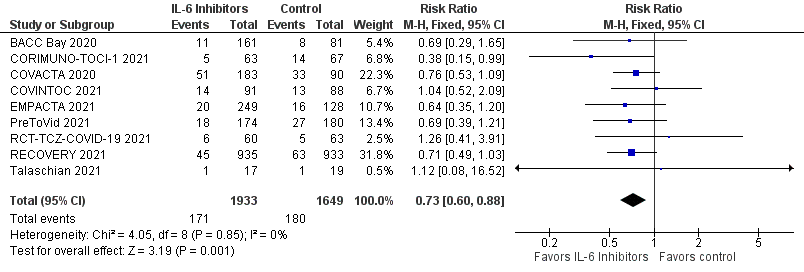


**eFigure 7 –** Forest plot for incidence of any serious adverse event


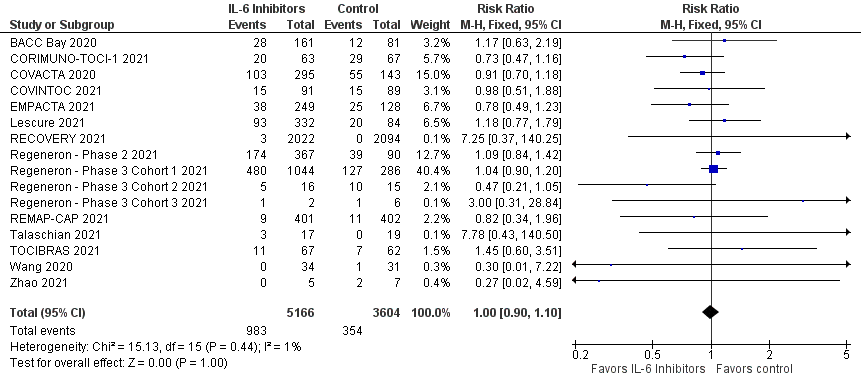


**eFigure 8 –** Forest plot for incidence of secondary infections


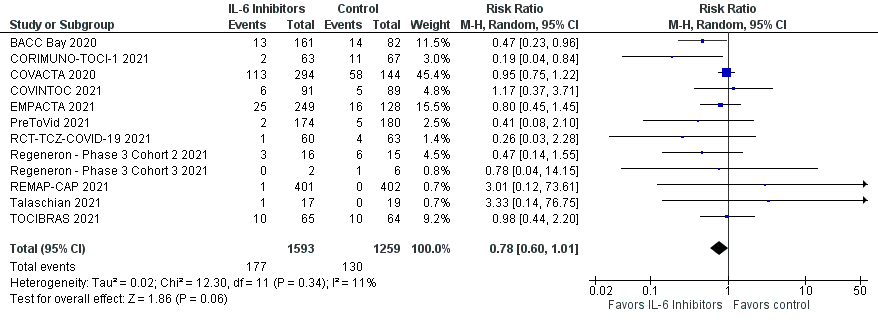


**eFigure 9 –** Forest plot for 28/30-days mortality, high vs low risk of bias studies


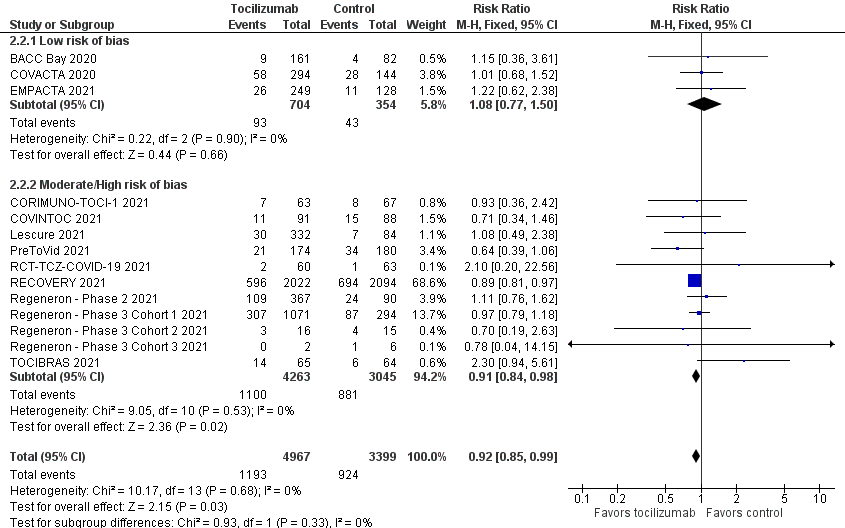


**eFigure 10 –** Forest plot for intubation, high vs low risk of bias studies.


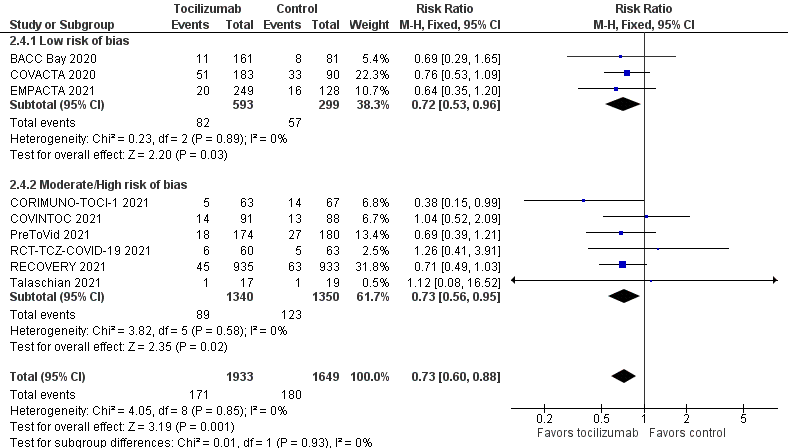


**eFigure 11 –** Forest plot for serious adverse events, high vs low risk of bias studies


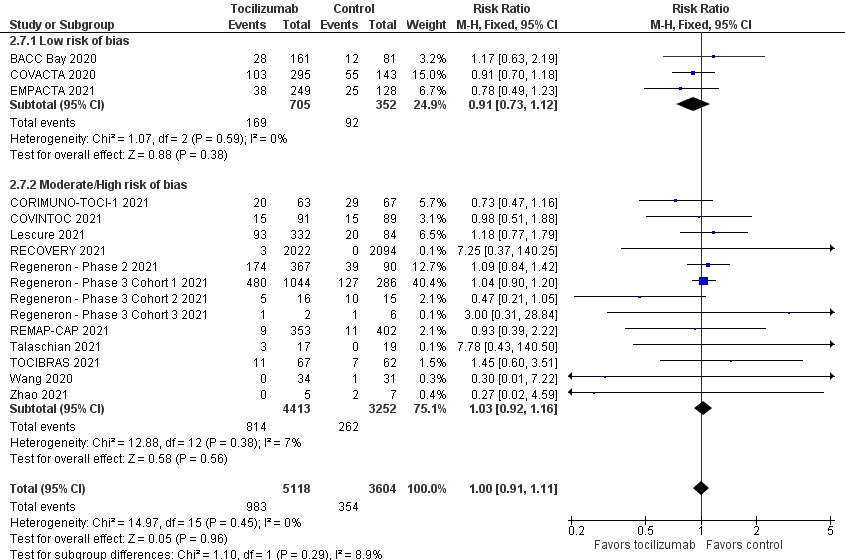


**eFigure 12 –** Forest plot for secondary infections, high vs low risk of bias studies


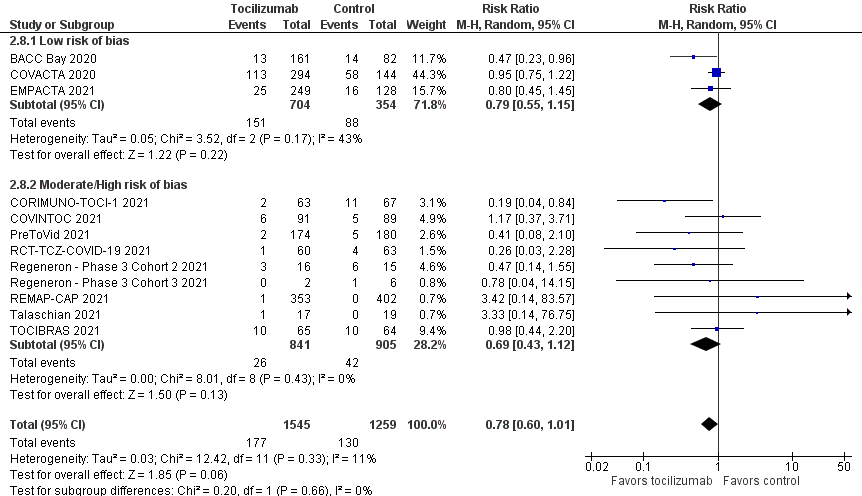


**eFigure 13 –** Forest plot for clinical worsening, high vs low risk of bias studies


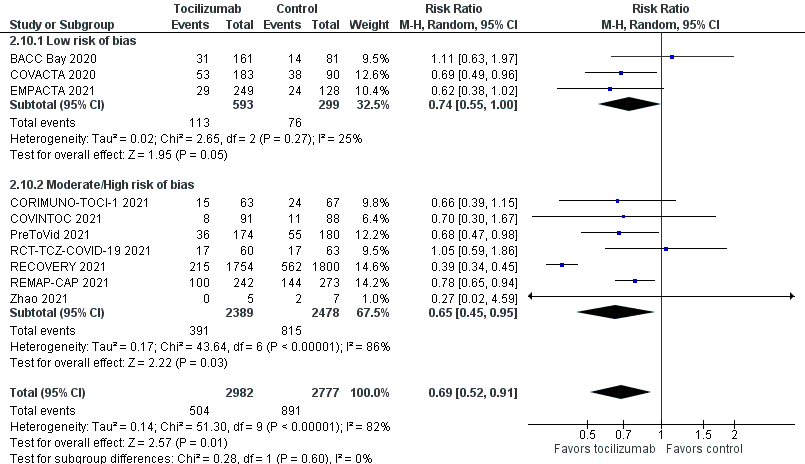


**eFigure 14 –** Forest plot longest follow-up mortality, studies enrolling vs not enrolling patients on IMV at baseline


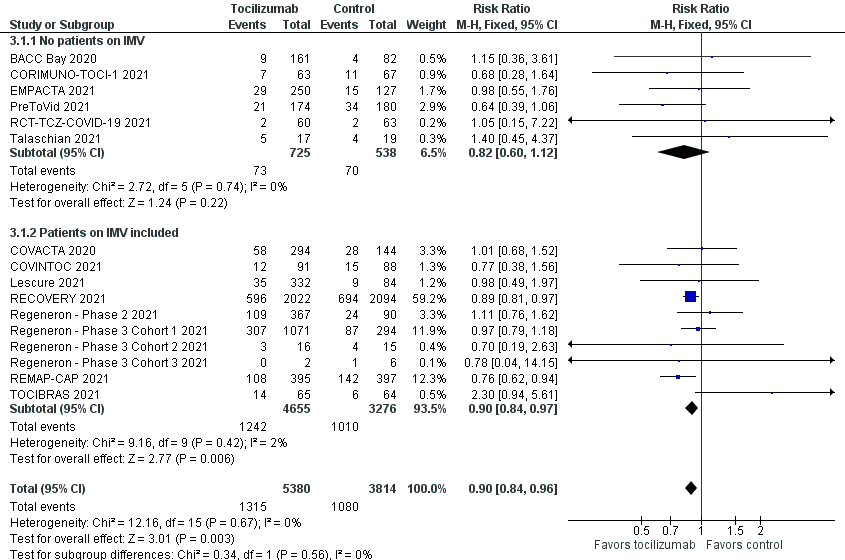


**eFigure 15 –** Forest plot 28/30-days mortality, studies enrolling vs not enrolling patients on IMV at baseline


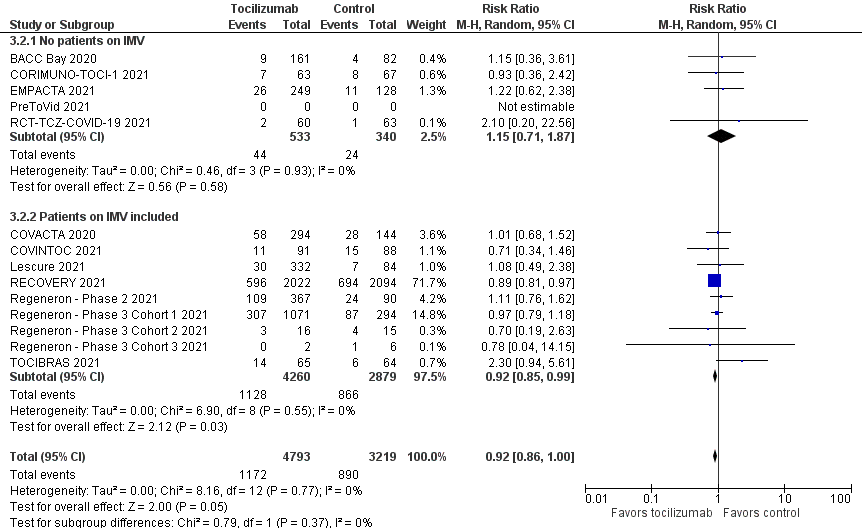


**eFigure 16 –** Forest plot for serious adverse events, studies enrolling vs not enrolling patients on IMV at baseline


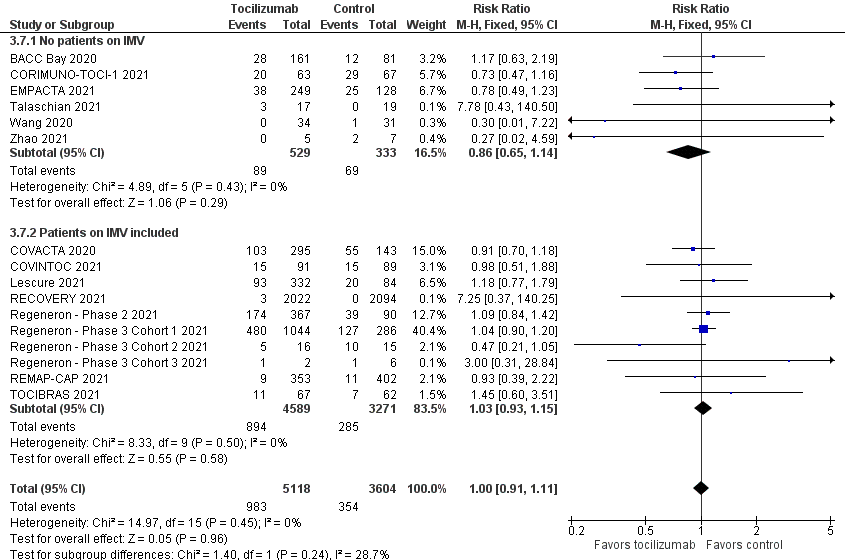


**eFigure 17 –** Forest plot for secondary infections, studies enrolling vs not enrolling patients on IMV at baseline


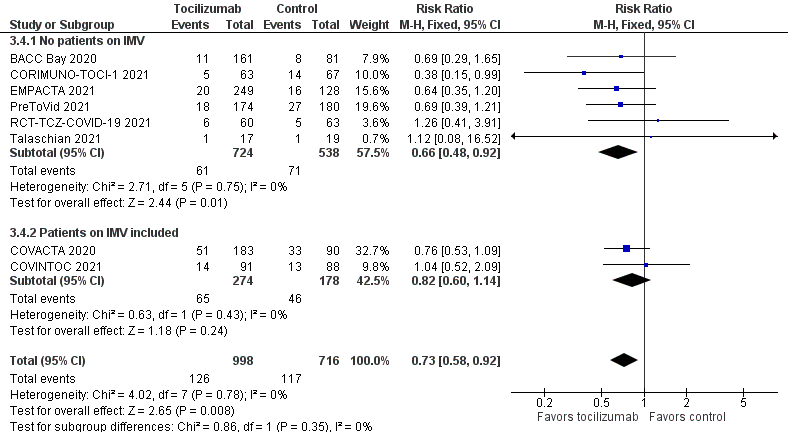


**eFigure 18 –** Forest plot for clinical worsening, studies enrolling vs not enrolling patients on IMV at baseline


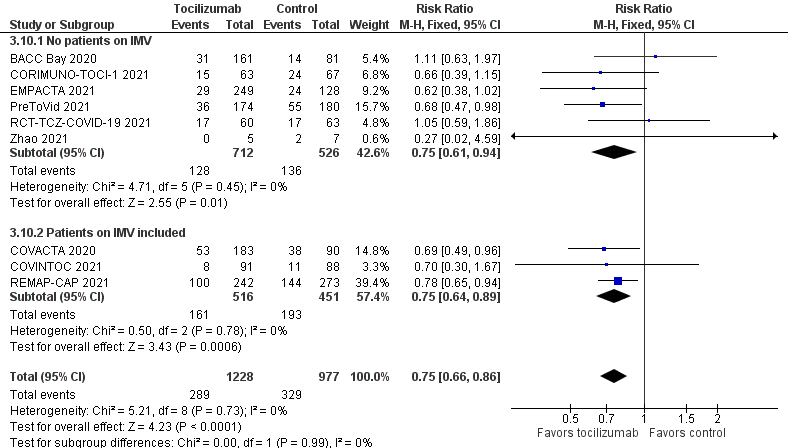


**eFigure 19 –** Forest plot for longest follow-up mortality, studies with high vs low prevalence of concomitant steroids use


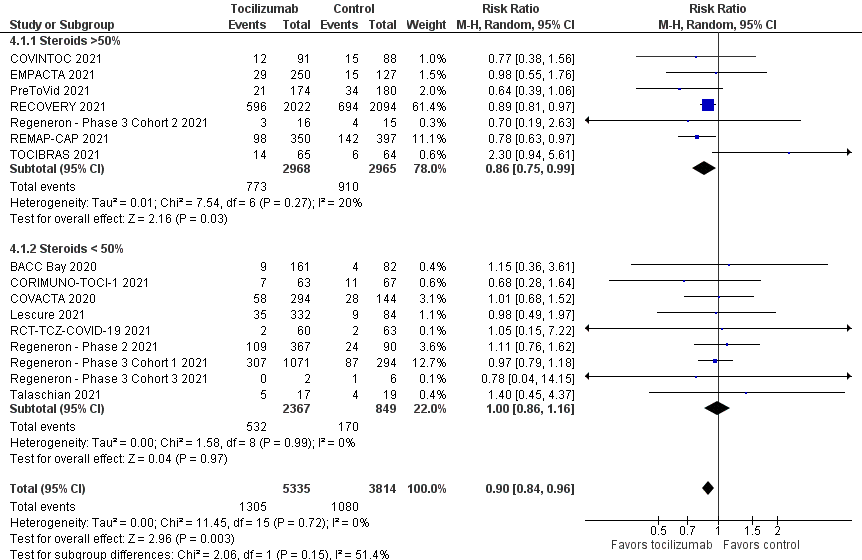


**eFigure 20 –** Forest plot for 28/30-days mortality, studies with high vs low prevalence of concomitant steroids use


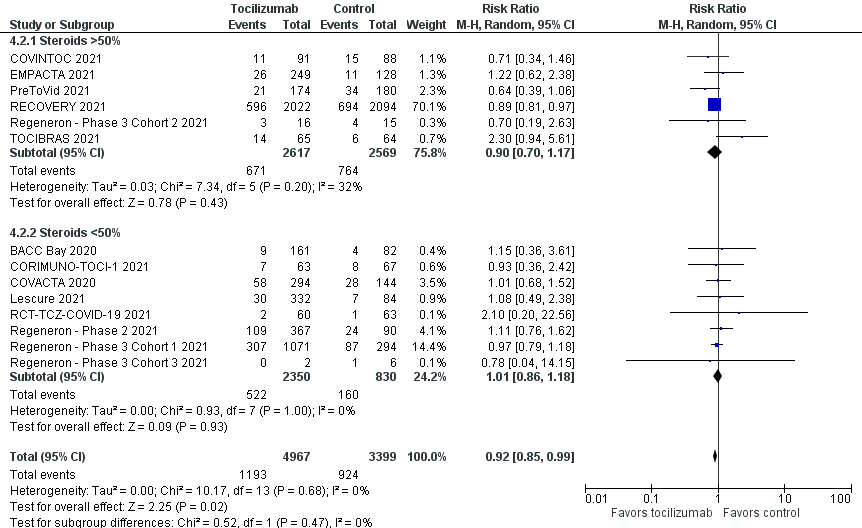


**eFigure 21 –** Forest plot for need for intubation, studies with high vs low prevalence of concomitant steroids use


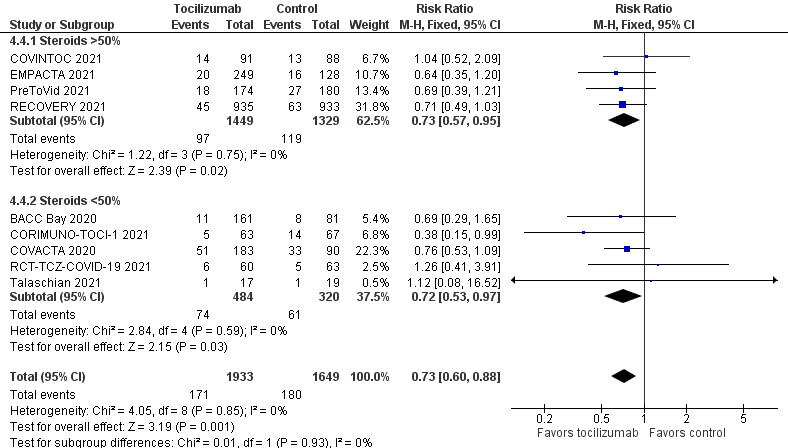


**eFigure 22 –** Forest plot for serious adverse events, studies with high vs low prevalence of concomitant steroids use


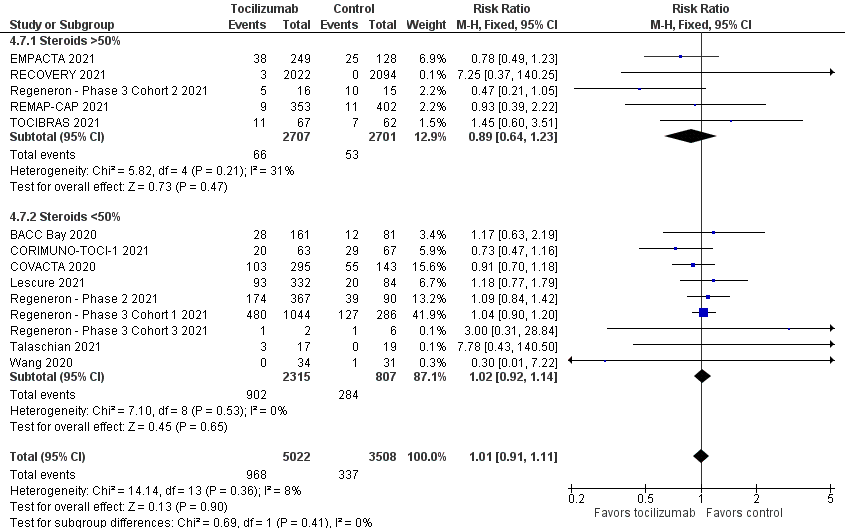


**eFigure 23 –** Forest plot for secondary infections, studies with high vs low prevalence of concomitant steroids use


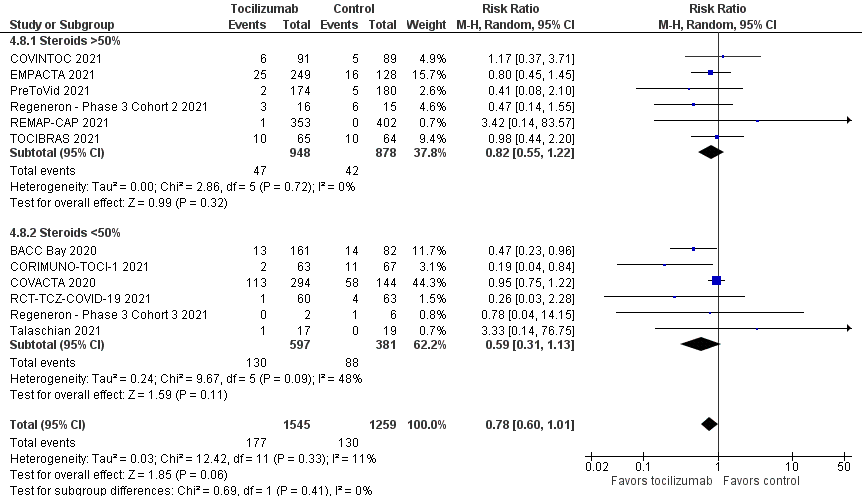


**eFigure 24 –** Forest plot for clinical worsening, studies with high vs low prevalence of concomitant steroids use


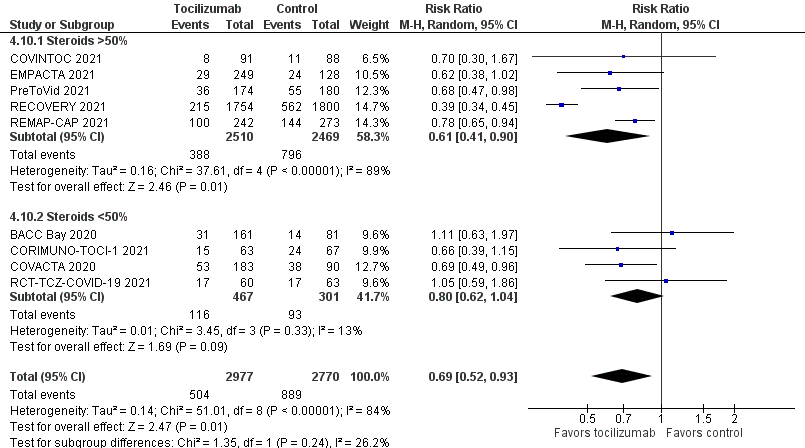


**eFigure 25 –** Forest plot for 28/30-days mortality, patients receiving versus those not receiving concomitant steroids administration


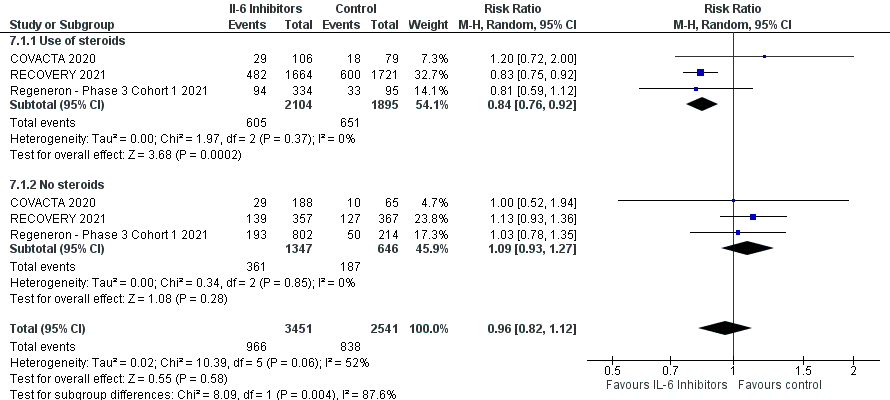

Supplement: Supplementary file 1 — Additional file 1. Supplementary Appendix including details on search strategies, supplementary tables S1–S2, and supplementary figures S1–S25. [file 13613_2021_941_MOESM1_ESM.doc]
